# Development and Evaluation of an Innovative Inflammatory Prognostic Score for Predicting Long‐Term Mortality in Patients With Pulmonary Embolism

**DOI:** 10.1155/mi/6325915

**Published:** 2025-12-22

**Authors:** Ning Zhu, Lei Zhang, Jiabao Wu, Fei Ye, Nanding Yu, Chao Cao, Limin Chen

**Affiliations:** ^1^ Department of Respiratory and Critical Care Medicine, Fujian Medical University Union Hospital, Fuzhou, Fujian, China, fjmu.edu.cn; ^2^ Department of Respiratory and Critical Care Medicine, The First Affiliated Hospital of Ningbo University, Ningbo, China, nbu.edu.cn; ^3^ Department of Respiratory and Critical Care Medicine, The Affiliated People Hospital of Ningbo University, Ningbo, China, nbu.edu.cn

**Keywords:** acute pulmonary embolism, all-cause mortality, biomarkers, inflammation, inflammatory prognostic scoring (IPS)

## Abstract

**Background:**

Systemic inflammation is closely associated with adverse outcomes in pulmonary embolism (PE). This study aimed to develop and evaluate a novel inflammatory prognostic score (IPS) derived from multiple inflammation‐related biomarkers to predict long‐term all‐cause mortality in patients with acute PE.

**Methods:**

This retrospective cohort study included 1642 patients with confirmed acute PE admitted between January 2016 and January 2024 at the First Affiliated Hospital of Ningbo University and the Affiliated People Hospital of Ningbo University. The primary outcome was all‐cause mortality. Follow‐up was conducted in January 2025. Fifteen inflammatory biomarkers were analyzed, including C‐reactive protein (CRP), white blood cell count (WBC), neutrophil count (NEU), lymphocyte count (LYM), monocyte count (MON), red cell distribution width (RDW), platelet count (PLT), and eight derived indices: neutrophil‐to‐lymphocyte ratio (NLR), derived NLR (dNLR), monocyte‐to‐lymphocyte ratio (MLR), neutrophil–MLR (NMLR), platelet‐to‐lymphocyte ratio (PLR), systemic immune‐inflammation index (SII), systemic inflammation response index (SIRI), and inflammatory burden index (IBI). IPS was constructed using least absolute shrinkage and selection operator (LASSO) Cox regression. Prognostic performance was assessed using Kaplan–Meier survival analysis, multivariable Cox regression, time‐dependent receiver operating characteristic (ROC) analysis, and random survival forest (RSF).

**Results:**

During a median follow‐up of 43.87 months, 262 patients (16.0%) died. The IPS was calculated as: IPS = 0.395 × IBI + 0.236 × RDW + 0.208 × SIRI + 0.115 × MLR + 0.040 × CRP. Patients with high IPS (≥0.55) had a significantly higher risk of all‐cause mortality compared to those with low IPS (adjusted hazard ratio [HR] = 2.55, 95% confidence intervals [CI]: 1.92–3.38; *p* < 0.001). Time‐dependent area under the curve (AUC) for IPS were 0.756, 0.768, and 0.773 at 1, 3, and 5 years, respectively. When added to the baseline clinical model—including age, length of hospital stay, deep vein thrombosis, diastolic blood pressure, body mass index (BMI), lactate, log‐transformed serum creatinine, blood urea nitrogen (BUN), log‐transformed D‐dimer, left ventricular ejection fraction (LVEF), pulmonary artery systolic pressure (PASP), and computed tomography pulmonary angiography (CTPA)‐based embolism location (main pulmonary artery)—IPS improved the AUC from 0.820 to 0.886 at 3 years (*p* < 0.001, DeLong test). RSF analysis further identified IPS as the most informative inflammatory predictor of mortality.

**Conclusions:**

The IPS, derived from five routinely available inflammatory biomarkers, was independently associated with long‐term mortality and significantly enhanced risk stratification beyond traditional clinical predictors in patients with acute PE. This score may support early prognostic assessment and individualized management.

## 1. Introduction

Pulmonary embolism (PE) is a common and life‐threatening cardiovascular disease characterized by the obstruction of the pulmonary arteries or their branches by thrombi, which typically originate from the deep venous system or the right side of the heart [1, 2]. The global data showed that the incidence of PE has been steadily increasing over the past two decades, with recent estimates ranging from 39 to 115 cases per 100,000 individuals annually [3]. Although considerable progress has been made in the diagnosis and treatment of PE, it remains a significant cause of mortality worldwide. PE is responsible for ~0.46% of all registered deaths, with age‐standardized mortality rates exhibiting notable international variation [4]. Notably, two European studies have reported 30‐day all‐cause mortality rates of up to 5.1% and 7.8% among individuals with nonhigh‐risk PE [5, 6]. Given its rising incidence, high mortality risk, and considerable socioeconomic impact, accurate prognostic assessment and risk stratification in patients with pulmonary PE are essential.

Nowadays, many prognostic models with varying levels of performance are used in clinical practice to identify patients with PE who are at low risk of adverse outcomes. Among the most widely endorsed are the PE Severity Index (PESI) and its simplified version (sPESI), both of which are recommended by the European Society of Cardiology (ESC) and the European Respiratory Society guidelines [7, 8]. In addition, a recently proposed model, the Peking Union Medical College Hospital (PUMCH) rule, has demonstrated superior discriminative power for predicting 30‐day mortality in PTE patients within a Chinese population cohort [9]. Although existing prognostic tools are effective in identifying low‐risk patients with PE, their utility in accurately stratifying high‐risk individuals remains limited due to relatively low specificity [10]. This limitation often necessitates the use of additional diagnostic modalities to confirm risk status, such as echocardiography or the 6‐minute walk test, which potentially increase the clinical burden and overall healthcare costs [10].

Accurate prognostication in acute PE depends on the identification of reliable and accessible biomarkers. Several sample biomarkers show promise as prognostic markers. Inflammation plays a crucial role in the pathophysiology and prognosis of PE [11]. Beyond mechanical obstruction of the pulmonary vasculature, PE induces a systemic inflammatory response characterized by leukocyte activation, endothelial dysfunction, and cytokine release [11]. In recent years, noninvasive inflammatory biomarkers have garnered attention as potential tools to assess disease severity and predict outcomes. Biomarkers such as C‐reactive protein (CRP), neutrophil‐to‐lymphocyte ratio (NLR), platelet‐to‐lymphocyte ratio (PLR), and systemic immune‐inflammation index (SII) have been investigated in various diseases, including PE [12]. Previous evidence has suggested that elevated NLR and PLR are promising inflammatory biomarkers for prognostication in acute PE [13]. While several studies have demonstrated the prognostic value of these indices in predicting mortality, some reports have yielded inconsistent or conflicting results [14, 15]. Moreover, complete blood count is a widely available, cost‐effective, and routinely performed laboratory test that provides valuable insights into a patient’s immune and inflammatory status. Therefore, the individual predictive utility of some indices remains inconsistent, and comprehensive approaches that integrate multiple markers may offer improved prognostic value.

There is a growing interest in utilizing inflammation‐related biomarkers to construct prognostic models for personalized risk assessment in patients with PE. Nonetheless, few studies have conducted analyses that simultaneously compare and integrate these biomarkers to evaluate their combined effectiveness in predicting all‐cause mortality among PE patients. The primary aim of this study was to develop and evaluate a novel inflammatory prognostic score (IPS) derived from multiple inflammation‐related biomarkers to predict long‐term all‐cause mortality in patients with acute PE. Additionally, the study sought to assess whether the IPS could improve risk stratification beyond existing clinical models and enhance individualized prognostic assessment.

## 2. Methods

### 2.1. Study Design and Participants

This retrospective cohort study included patients diagnosed with acute PE between January 2016 and January 2024 at the First Affiliated Hospital of Ningbo University and the Affiliated People Hospital of Ningbo University. All participants were admitted to general wards, and none received intensive care unit–level interventions during the early hospitalization period. PE was confirmed in all cases by computed tomography pulmonary angiography (CTPA). Inclusion criteria were: (1) age ≥ 18 years; (2) confirmed diagnosis of acute PE; (3) availability of baseline inflammatory biomarker data within 24 h of admission; and (4) complete clinical and follow‐up information. Exclusion criteria included: (1) severe hepatic or renal dysfunction, defined as alanine aminotransferase (ALT) or aspartate aminotransferase (AST) >5 times the upper limit of normal, or estimated glomerular filtration rate (eGFR) <15 mL/min/1.73 m^2^; (2) insufficient follow‐up data.

A total of 1642 patients met the eligibility criteria and were included in the final analysis. The primary endpoint was all‐cause mortality. Follow‐up was conducted in January 2025 through review of electronic medical records and telephone interviews with family members. All endpoint events were verified by clinical staff. The study protocol was approved by the Independent Ethics Committee of the First Affiliated Hospital of Ningbo University (No. 2024‐005RS) and the Affiliated People Hospital of Ningbo University (No. 2025‐027).

#### 2.1.1. Data Collection

Baseline demographic, clinical, laboratory, imaging, and treatment data were retrospectively extracted from the electronic medical records of all included patients. Variables collected at admission included age, sex, and length of hospital stay. Medical history encompassed hypertension, diabetes mellitus, coronary heart disease, heart failure, malignant tumor, history of fracture, and deep vein thrombosis. Vital signs and physical examination parameters included average heart rate, systolic and diastolic blood pressure, and body mass index (BMI). Arterial blood gas measurements within 24 h of admission included arterial pH, partial pressures of oxygen (PaO_2_) and carbon dioxide (PaCO_2_), bicarbonate (HCO_3_^−^), and lactate. Laboratory tests comprised serum creatinine, blood urea nitrogen (BUN), ALT, AST, D‐dimer, cardiac troponin T, and N‐terminal pro‐brain natriuretic peptide (NT‐proBNP). Transthoracic echocardiographic parameters included left ventricular end‐systolic diameter (LVDs), left ventricular end‐diastolic diameter (LVDd), left atrial diameter (LA), left ventricular ejection fraction (LVEF), and pulmonary artery systolic pressure (PASP). The location of PE was determined by CTPA and classified as involving the main pulmonary artery, main branch, upper lobe, lower lobe, bilateral, or small branches. Information on prior medication use, including thrombolytics, anticoagulants, and diuretics, was also recorded.

### 2.2. Inflammatory Biomarker Measurement and Derivation

Inflammatory biomarker data were obtained from blood samples collected within 24 h of admission. The biomarkers assessed included (CRP, mg/L), white blood cell count (WBC, 10^9^/L), neutrophil count (NEU, 10^9^/L), lymphocyte count (LYM, 10^9^/L), monocyte count (MON, 10^9^/L), red cell distribution width (RDW, %), and platelet count (PLT, 10^9^/L). In addition to individual parameters, several composite inflammatory indices were calculated to reflect systemic inflammatory status:

NLR=NEU/LYM.

Derivedneutrophil-to-lymphocyteratiodNLR=NEU/WBC−NEU.

Monocyte-to-lymphocyteratioMLR=MON/LYM.

Neutrophil−monocyte-to-lymphocyteratioNMLR=NEU+MON/LYM.

PLR=PLT/LYM.

SII=PLT×NEU/LYM.

Systemic inflammation response indexSIRI=NEU×MON/LYM.

Inflammatory burden index (IBI) was calculated as: CRP(mg/L) + [WBC(10^9^/L) × 10].

### 2.3. Development of Inflammatory Prognostic Scoring System

Given that the median follow‐up duration in this cohort was ~3.7 years, 36 months was selected as the time point for prognostic assessment. A time‐dependent receiver operating characteristic (ROC) curve was generated using the R package “timeROC” to evaluate the predictive value of 15 inflammation‐related biomarkers for all‐cause mortality. The optimal cutoff value for each biomarker was determined based on the maximum Youden index at the 3‐year time point, and all biomarkers were subsequently dichotomized into categorical variables accordingly.

To address multicollinearity and reduce dimensionality, least absolute shrinkage and selection operator (LASSO) Cox regression was performed using the R package “glmnet” with five‐fold cross‐validation. The optimal penalty parameter (*λ*) was selected based on the model with the highest concordance index (C‐index) within one standard error of the maximum. Inflammatory biomarkers with nonzero coefficients at this optimal *λ* value were retained to construct the IPS. The IPS was calculated as a linear combination of the selected biomarkers weighted by their respective LASSO‐derived coefficients: IPS = ∑ (biomarker value × corresponding coefficient).

The IPS was analyzed as both a continuous and categorical variable. Patients were stratified into high‐ and low‐risk groups based on the optimal IPS cutoff value identified through 3‐year ROC analysis. The prognostic utility of the IPS was further evaluated using survival analysis and multivariable Cox regression models.

### 2.4. Statistical Analysis

Continuous variables were presented as mean ± standard deviation (SD) or median with interquartile range (IQR), as appropriate, and compared using the independent samples *t*‐test or Mann–Whitney U test. Categorical variables were expressed as frequencies and percentages and compared using the chi‐square test or Fisher’s exact test, as appropriate. Spearman rank correlation analysis was performed to assess pairwise correlations among inflammatory biomarkers.

Time‐to‐event data were analyzed using Kaplan–Meier survival curves, and differences between groups were assessed using the log‐rank test. Cox proportional hazards regression was used to estimate hazard ratios (HRs) and 95% confidence intervals (CIs) for all‐cause mortality. A stepwise forward selection method was applied to identify independent predictors for inclusion in the multivariable model. Variables considered for entry into the model were based on clinical relevance and univariate significance (*p* < 0.10). The discriminative performance of the inflammation biomarkers was assessed using time‐dependent ROC curve analysis at 1, 3, and 5 years, with the area under the curve (AUC) calculated using the “timeROC” package. The incremental predictive value of IPS beyond the basic clinical model was evaluated by comparing AUCs using the DeLong test.

Random survival forest (RSF) analysis was performed to explore the relative importance of each inflammatory biomarker in predicting all‐cause mortality. Two metrics were used to assess variable importance (VIMP) within the RSF model. The first was VIMP, which quantifies the increase in prediction error caused by random permutation of a given variable, thereby reflecting its contribution to model accuracy. The second was minimal depth, defined as the average depth of the first split for each variable across all decision trees; variables with smaller minimal depth values are considered more predictive as they tend to appear closer to the root node of the trees. Variables with higher VIMP and lower minimal depth were considered more predictive. The RSF model was built using 1000 trees and default settings in the “randomForestSRC” package.

To assess the robustness of our findings, two prespecified sensitivity analyses were performed. First, we excluded participants with a documented history of malignancy. Second, we excluded those meeting any criterion of the inflammatory disease set. In both restricted cohorts, we repeated the primary multivariable Cox regression models using the same covariates as in the main analysis. All statistical analyses were conducted using R software (Version 4.3.1), and a two‐sided *p* value < 0.05 was considered statistically significant.

## 3. Results

### 3.1. Baseline Characteristics

A total of 1642 patients with PE were included in the final analysis, with a median follow‐up duration of 43.87 months, during which 262 patients (16.0%) died. The baseline characteristics of the study population are presented in Table 1. Compared with survivors, nonsurvivors were significantly older and more likely to be male, with longer hospital stays and a higher prevalence of comorbidities, including hypertension, coronary heart disease, heart failure, and deep vein thrombosis (all *p* < 0.01). Inflammatory markers were notably elevated in patients who died. Levels of CRP, WBC, NEU, MON, RDW, and composite indices such as NLR, derived NLR (dNLR), monocyte‐to‐lymphocyte ratio (MLR), neutrophil–monocyte‐to‐lymphocyte ratio (NMLR), SII, systemic inflammation response index (SIRI), and IBI were all significantly higher in the death group, while LYM and PLT were lower.

**Table 1:** Baseline characteristics in patients with pulmonary embolism.

Characteristics	Total (*n* = 1642)	Survival (*n* = 1380)	Death (*n* = 262)	*p* value
Age, years	54.40 ± 12.76	52.77 ± 12.51	62.99 ± 10.47	<0.001
Male (%)	832 (50.7%)	674 (48.8%)	158 (60.3%)	0.001
Length of hospital stay (days)	15.02 ± 17.54	14.06 ± 15.66)	20.09 ± 24.64	<0.001
Medical history (%)				
Hypertension	775 (47.2%)	618 (44.8%)	157 (59.9%)	<0.001
Diabetes mellitus	174 (10.6%)	137 (9.9%)	37 (14.1%)	0.056
Coronary heart disease	183 (11.1%)	132 (9.6%)	51 (19.5%)	<0.001
Heart failure	64 (3.9%)	40 (2.9%)	24 (9.2%)	<0.001
Malignant tumor	147 (9.0%)	120 (8.7%)	27 (10.3%)	0.472
History of fracture	120 (7.3%)	94 (6.8%)	26 (9.9%)	0.100
Deep vein thrombosis	94 (5.7%)	58 (4.2%)	36 (13.7%)	<0.001
Physical examination				
Average heart rate (bpm)	75.85 ± 16.82	75.34 ± 16.64	78.54 ± 17.53	0.005
Systolic BP (mmHg)	128.85 ± 20.58	129.81 ± 20.14	123.81 ± 22.17	<0.001
Diastolic BP (mmHg)	76.47 ± 12.46	77.44 ± 12.19	71.36 ± 12.64	<0.001
BMI (kg/m^2^)	23.92 ± 3.89	24.06 ± 3.91	23.15 ± 3.70	0.001
Arterial blood gas				
Arterial pH	7.42 ± 0.06	7.42 ± 0.05	7.42 ± 0.07	0.289
PaO_2_ (mmHg)	81.66 ± 23.05	82.25 ± 22.66	78.56 ± 24.83	0.018
PaCO_2_ (mmHg)	38.73 ± 8.00	38.91 ± 7.55	37.80 ± 10.02	0.039
HCO_3_ (mmol/L)	25.26 ± 4.48	25.41 ± 4.16	24.48 ± 5.83	0.002
Lactate (mmol/L)	1.83 ± 1.48	1.77 ± 1.39	2.12 ± 1.88	<0.001
Laboratory measures				
Serum creatinine (µmol/L)	70.00 [59.00, 87.00]	68.00 [58.00, 82.00]	87.07 [70.00, 118.78]	<0.001
BUN (mmol/L)	5.55 [4.38, 7.20]	5.33 [4.24, 6.65]	7.85 [5.72, 10.75]	<0.001
ALT (U/L)	20.00 [13.00, 34.74]	19.16 [13.28, 33.00]	22.00 [13.00, 51.75]	0.011
AST (U/L)	24.00 [19.00, 37.00]	23.41 [18.00, 34.00]	27.12 [20.00, 59.00]	<0.001
D‐dimer (ng/mL)	1491.27 [464.50, 3455.75]	1203.50 [392.50, 3180.53]	2328.50 [875.50, 3680.00]	<0.001
Troponin T (ng/mL)	0.03 [0.02, 0.10]	0.03 [0.01, 0.09]	0.06 [0.03, 0.24]	<0.001
NT‐proBNP (ng/L)	589.98 [250.33, 1611.73]	522.60 [224.61, 1292.27]	1388.01 [556.93, 3881.85]	<0.001
Inflammatory biomarkers				
CRP (mg/L)	10.38 [1.60, 37.92]	7.68 [1.24, 31.46]	26.96 [9.91, 64.57]	<0.001
WBC (10^9^/L)	6.78 [5.17, 9.10]	6.60 [5.03, 8.69]	7.83 [5.93, 10.97]	<0.001
NEU (10^9^/L)	4.55 [3.20, 6.90]	4.30 [3.10, 6.50]	6.00 [3.90, 8.97]	<0.001
LYM (10^9^/L)	1.30 [0.90, 1.70]	1.30 [0.90, 1.80]	1.00 [0.70, 1.58]	<0.001
MON (10^9^/L)	0.50 [0.40, 0.70]	0.50 [0.38, 0.62]	0.60 [0.40, 0.80]	<0.001
RDW (%)	13.20 [12.50, 14.30]	13.10 [12.40, 14.00]	13.90 [13.00, 15.30]	<0.001
PLT (10^9^/L)	202.00 [155.25, 255.75]	205.00 [160.00, 258.25]	182.00 [138.00, 243.00]	<0.001
IBI	35.29 [4.56, 195.44]	25.03 [3.13, 143.77]	142.37 [33.97, 516.23]	<0.001
NLR	3.54 [2.22, 6.49]	3.27 [2.11, 5.86]	5.28 [3.26, 10.37]	<0.001
dNLR	2.33 [1.55, 3.78]	2.21 [1.48, 3.46]	3.22 [2.09, 5.55]	<0.001
MLR	0.39 [0.27, 0.61]	0.37 [0.26, 0.57]	0.54 [0.38, 0.83]	<0.001
NMLR	3.94 [2.55, 7.11]	3.67 [2.41, 6.44]	5.87 [3.63, 11.09]	<0.001
PLR	160.00 [112.50, 235.20]	157.27 [112.92, 228.90]	177.00 [106.91, 288.86]	0.044
SII (10^9^/L)	701.62 [416.79, 1294.89]	667.14 [408.52, 1200.35]	962.41 [531.20, 1952.14]	<0.001
SIRI (10^9^/L)	1.80 [0.94, 3.83]	1.60 [0.88, 3.32]	3.18 [1.67, 6.38]	<0.001
Transthoracic echocardiography				
LVDs	30.17 ± 3.69	29.82 ± 3.24	32.02 ± 5.12	<0.001
LVDd	46.45 ± 3.93	46.19 ± 3.59	47.83 ± 5.17	<0.001
LA	35.73 ± 4.14	35.24 ± 3.91	38.28 ± 4.39	<0.001
LVEF	62.45 ± 6.58	63.57 ± 5.34	56.58 ± 8.97	<0.001
PASP	37.55 ± 8.71	36.81 ± 8.56	41.44 ± 8.47	<0.001
CTPA‐based embolism location				
Main pulmonary artery	234 (14.3%)	169 (12.2%)	65 (24.8%)	<0.001
Main branch	839 (51.1%)	700 (50.7%)	139 (53.1%)	0.533
Upper lobe	209 (12.7%)	181 (13.1%)	28 (10.7%)	0.327
Lower lobe	545 (33.2%)	441 (32.0%)	104 (39.7%)	0.018
Bilateral	57 (3.5%)	45 (3.3%)	12 (4.6%)	0.376
Small branch	20 (1.2%)	17 (1.2%)	3 (1.1%)	0.990
Prior medication (%)				
Thrombolysis	163 (9.9%)	144 (10.4%)	19 (7.3%)	0.142
Anticoagulants	1570 (95.6%)	1319 (95.6%)	251 (95.8%)	0.991
Diuretics	655 (39.9%)	478 (34.6%)	177 (67.6%)	<0.001
Follow‐up duration (months)	43.87 ± 27.25	48.85 ± 25.06	17.66 ± 23.03	<0.001

*Note*: Continuous variables are expressed as mean ± SD or as median [interquartile range] and categorical variables are expressed as number (%). ALT, alanine aminotransferase; AST, aspartate aminotransferase; cTnI, cardiac troponin I; LVDd, left ventricular end‐diastolic diameter; LVDs, left ventricular end‐systolic diameter; LYM, lymphocyte count; MON, monocyte count; NEU, neutrophil count; NT‐proBNP,N‐terminal pro–B‐type natriuretic peptide; PaCO_2_, partial pressure of arterial carbon dioxide; PaO_2_, partial pressure of arterial oxygen; PLT, platelet count.

Abbreviations: BMI, body mass index; BP, blood pressure; BUN, blood urea nitrogen; CRP, C‐reactive protein; CTPA, computed tomography pulmonary angiography; dNLR, derived neutrophil‐to‐lymphocyte ratio; HCO_3_^-^, bicarbonate; IBI, inflammatory burden index; LA, left atrial diameter; LVEF, left ventricular ejection fraction; MLR, monocyte‐to‐lymphocyte ratio;NLR, neutrophil‐to‐lymphocyte ratio; NMLR, neutrophil–monocyte‐to‐lymphocyte ratio; PASP, pulmonary artery systolic pressure; PLR, platelet‐to‐lymphocyte ratio; RDW, red cell distribution width; SII, systemic immune‐inflammation index; SIRI, systemic inflammation response index; WBC, white blood cell count.

Nonsurvivors exhibited more pronounced hemodynamic compromise, including higher heart rate, lower systolic and diastolic blood pressure, and elevated lactate levels. In terms of organ function, they had significantly worse renal parameters (serum creatinine and BUN), elevated liver enzymes (ALTand AST), and higher cardiac biomarker levels (troponin T and NT‐proBNP) (all *p* < 0.01). Echocardiographic findings showed that nonsurvivors had larger left ventricular and atrial diameters, lower LVEF, and higher PASP, suggesting more severe cardiac involvement. Moreover, emboli involving the main pulmonary artery and lower lobes were more common in nonsurvivors. Use of diuretics was significantly more frequent in the death group, although the use of thrombolytics and anticoagulants did not differ between groups.

### 3.2. Prognostic Stratification of Inflammatory Biomarkers Based on Optimal Cutoff Values

To explore the prognostic value of inflammation‐related biomarkers, time‐dependent ROC curve analysis was performed for 15 inflammatory indicators at 1‐, 3‐, and 5‐year time points (Supporting Information: Figure S1). Given the median follow‐up duration of 43.87 months, the optimal cutoff values for each biomarker were determined based on the maximum Youden index at the 3‐year time point (Table 2). Using the derived optimal thresholds, patients were categorized into high and low biomarker groups. Kaplan–Meier survival curves revealed significant differences in all‐cause mortality between groups for each biomarker (Supporting Information: Figure S2; all log‐rank *p* < 0.001).

**Table 2:** Area under the curve (AUC) and optimal threshold for inflammatory biomarkers to predict 3‐year all‐cause mortality in patients with pulmonary embolism.

Variables	AUC (95% CI)	Cutoff value	Sensitivity	Specificity
CRP (mg/L)	0.702 (0.665–0.740)	12.73	0.735	0.606
WBC (10^9^/L)	0.641 (0.600–0.686)	8.91	0.456	0.792
NEU (10^9^/L)	0.672 (0.630–0.714)	5.00	0.634	0.655
LYM (10^9^/L)	0.636 (0.591–0.682)	1.10	0.406	0.358
MON (10^9^/L)	0.618 (0.573–0.664)	0.55	0.606	0.640
RDW (%)	0.680 (0.640–0.719)	13.70	0.573	0.717
PLT (10^9^/L)	0.581 (0.535–0.626)	159.00	0.588	0.240
IBI (mg/L)	0.737 (0.702–0.772)	26.14	0.706	0.664
NLR	0.705 (0.666–0.745)	5.40	0.533	0.780
dNLR	0.685 (0.644–0.726)	2.61	0.649	0.644
MLR	0.697 (0.656–0.738)	0.49	0.608	0.699
NMLR	0.709 (0.669–0.748)	5.83	0.542	0.770
PLR	0.564 (0.516–0.612)	267.14	0.304	0.853
SII (10^9^/L)	0.637 (0.593–0.681)	978.09	0.521	0.716
SIRI (10^9^/L)	0.713 (0.674–0.753)	2.49	0.641	0.706

*Note:* LYM, lymphocyte count; MON, monocyte count; NEU, neutrophil count; PLT, platele tcount.

Abbreviations: CRP, C‐reactive protein; dNLR, derived neutrophil‐to‐lymphocyte ratio; IBI, inflammatory burden index; MLR, monocyte‐to‐lymphocyte ratio; NLR, neutrophil‐to‐lymphocyte ratio; NMLR, neutrophil‐monocyte‐to‐lymphocyte ratio; PLR, platelet‐to‐lymphocyte ratio; PLT, platelet RDW, red cell distribution width; SII, systemic immune‐inflammation index; SIRI, systemic inflammation response index; WBC, white blood cell count.

### 3.3. Development of IPS and Its Predictive Value for Mortality

Pairwise correlations among the 15 inflammatory biomarkers were assessed using Spearman correlation analysis (Supporting Information: Figure S3). Strong positive correlations were observed among composite indices such as NLR, dNLR, NMLR, and SIRI, indicating potential multicollinearity in predictive modeling.

To construct a parsimonious inflammatory prognostic scoring system, all 15 biomarkers were subjected to LASSO‐Cox regression. As shown in Figure 1A, the coefficient profiles of the candidate biomarkers were progressively shrunk with increasing regularization. Ten‐fold cross‐validation was used to determine the optimal *λ* value that achieved the highest mean C‐index within one standard error (Figure 1B). Based on this selection, five biomarkers with nonzero coefficients were retained in the final model: IBI, RDW, SIRI, MLR, and CRP (Figure 1C). The IPS was subsequently calculated as follows: IPS = 0.395 × IBI + 0.236 × RDW + 0.208 × SIRI + 0.115 × MLR + 0.040 × CRP. Using the maximum Youden index at the 3‐year time point, the optimal IPS cutoff value was determined to be 0.55. Patients were stratified into a low IPS group (<0.55) and a high IPS group (≥0.55).

Figure 1Development of the Inflammatory Prognostic Score (IPS) using LASSO regression analysis. (a) Coefficient profile plot generated from LASSO regression analysis of 15 candidate inflammatory biomarkers. Each colored line represents the trajectory of a variable’s coefficient as a function of the regularization parameter *λ* (log scale). As the penalty increases (left to right), more coefficients shrink toward zero. (b) Ten‐fold cross‐validation plot showing the mean C‐index for different values of log(*λ*). The left dotted line indicates the *λ* value with the minimum cross‐validated error (optimal *λ*), and the right dotted line represents the largest *λ* within one standard error of the minimum (simplest model). (c) Schematic diagram illustrating the stepwise selection and integration process for constructing the IPS. Among the 12 inflammatory markers, 5 variables (IBI, RDW, SIRI, MLR, and CRP) were selected by the LASSO model. The final IPS was calculated as: IPS = 0.395 × IBI + 0.236 × RDW + 0.208 × SIRI + 0.115 × MLR + 0.040 × CRP. Patients were stratified into two groups based on the IPS cut‐off value of 0.55: low IPS group (<0.55, *n* = 958) and high IPS group (≥0.55, *n* = 684).

**Figure:**
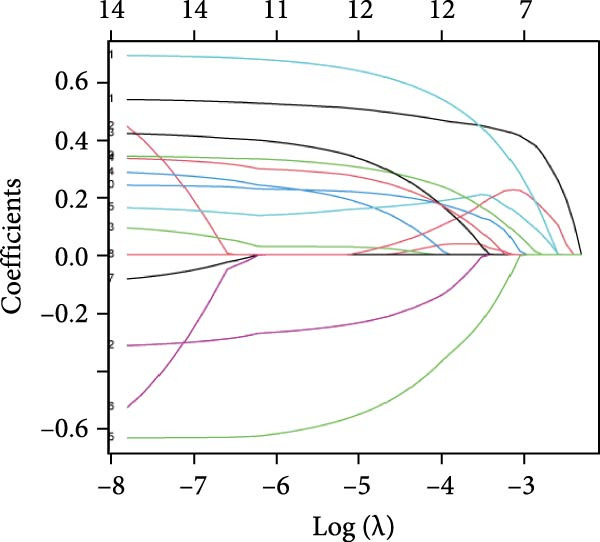
(a)

**Figure:**
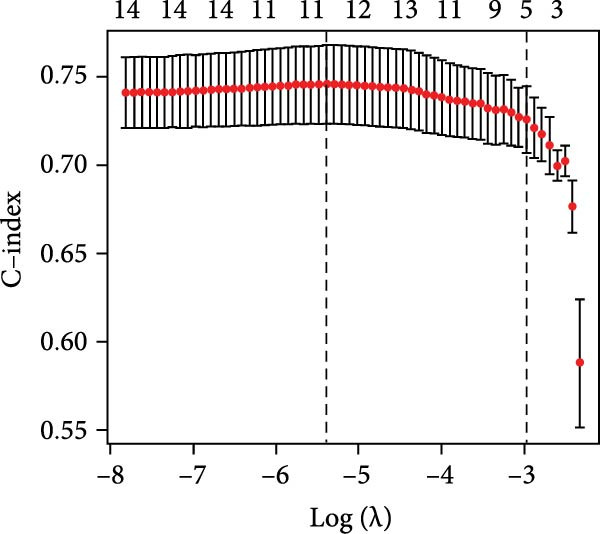
(b)

**Figure:**
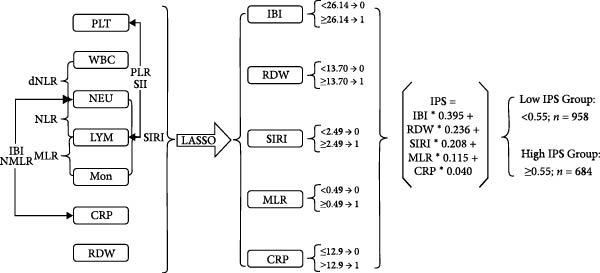
(c)

Time‐dependent ROC curve analysis demonstrated good discrimination of the IPS for predicting all‐cause mortality, with AUCs of 0.756, 0.768, and 0.773 at 1, 3, and 5 years, respectively (Figure 2A). Kaplan–Meier analysis revealed significantly reduced survival in the high IPS group compared to the low IPS group (log‐rank *p* < 0.001; Figure 2B).

Figure 2Prognostic value of the Inflammatory Prognostic Score (IPS) for all‐cause mortality. (a) Time‐dependent ROC curves showing the discriminative performance of IPS for 1‐, 3‐, and 5‐year all‐cause mortality. The optimal cutoff value (0.55) was selected at the 3‐year time point based on the maximum Youden index, considering that the mean follow‐up duration was ~43.87 ± 27.25 months. (b) Kaplan–Meier survival curves stratified by IPS levels. Patients with high IPS (≥0.55) had significantly poorer survival compared to those with low IPS (<0.55) (*p* < 0.001, log‐rank test). The adjusted hazard ratio (HR) was derived from a multivariable Cox regression model adjusted for age, length of hospital stay, deep vein thrombosis, diastolic blood pressure, body mass index, lactate, log‐transformed serum creatinine, blood urea nitrogen, log‐transformed D‐dimer, left ventricular ejection fraction, pulmonary artery systolic pressure, and CTPA‐based embolism location (main pulmonary artery).

**Figure:**
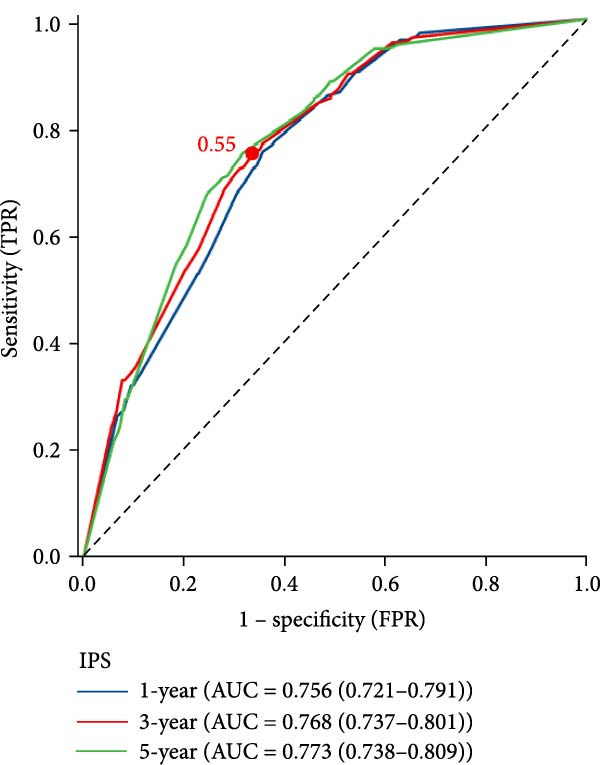
(a)

**Figure:**
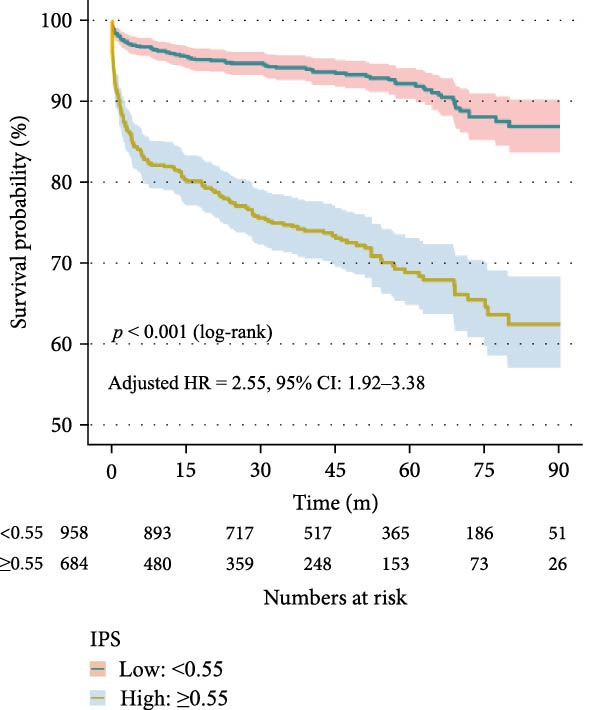
(b)

### 3.4. Independent Associations of Inflammatory Markers With All‐Cause Mortality

Univariate Cox regression identified multiple clinical variables associated with all‐cause mortality (Table 3). Variables with *p* < 0.05 were entered into a multivariate Cox model using stepwise forward selection. The final model retained the following as independent predictors: age, length of hospital stay, deep vein thrombosis, diastolic blood pressure, BMI, lactate, log‐transformed serum creatinine, BUN, log‐transformed D‐dimer, LVEF, PASP, and main pulmonary artery involvement. These variables were collectively defined as the clinical baseline model.

**Table 3:** Univariate and multivariate stepwise Cox regression analysis for all‐cause mortality in patients with pulmonary embolism.

Variables	Univariate analysis		Multivariate analysis	
	HR (95%CI)	*p* value	HR (95% CI)	*p* value
Age	1.10 (1.08–1.11)	<0.001	1.11 (1.09–1.13)	<0.001
Male	1.58 (1.23–2.02)	<0.001	—	—
Length of hospital stay	1.01 (1.01–1.02)	<0.001	1.02 (1.02–1.02)	<0.001
Hypertension	1.82 (1.42–2.33)	<0.001	—	—
DM	1.49 (1.05–2.11)	0.026	—	—
CHD	2.21 (1.63–3.00)	<0.001	—	—
Heart failure	4.02 (2.64–6.13)	<0.001	—	—
History of fracture	1.53 (1.02–2.29)	0.040	—	—
Deep vein thrombosis	2.63 (1.85–3.74)	<0.001	1.95 (1.36–2.81)	<0.001
Average heart rate	1.01 (1.00–1.02)	0.002	—	—
Systolic BP	0.99 (0.98–0.99)	<0.001	—	—
Diastolic BP	0.96 (0.95–0.97)	<0.001	0.98 (0.97–0.99)	<0.001
BMI	0.94 (0.91–0.97)	<0.001	0.96 (0.93–1.00)	0.026
PaO_2_	0.99 (0.98–0.99)	0.005	—	—
Lactate	1.12 (1.07–1.18)	<0.001	1.08 (1.01–1.16)	0.028
Ln Serum creatinine	8.51 (6.69–10.82)	<0.001	2.57 (1.70–3.89)	<0.001
BUN	1.19 (1.17–1.21)	<0.001	1.08 (1.05–1.11)	<0.001
Ln ALT	1.47 (1.29–1.68)	<0.001	—	—
Ln AST	1.77 (1.57–2.00)	<0.001	—	—
Ln D‐dimer	1.42 (1.29–1.57)	<0.001	1.27 (1.14–1.41)	<0.001
LncTnI	1.27 (1.19–1.36)	<0.001	—	—
Ln NT‐proBNP	1.89 (1.71–2.09)	<0.001	—	—
LVEF	0.90 (0.89–0.91)	<0.001	0.88 (0.87–0.89)	<0.001
PASP	1.04 (1.03–1.05)	<0.001	1.02 (1.01–1.04)	<0.001
Main pulmonary artery	2.13 (1.61–2.82)	<0.001	2.32 (1.72–3.13)	<0.001

*Note:* Variables with *p* < 0.05 in univariate analysis were entered into the multivariate Cox proportional hazards model using a stepwise forward selection approach. Continuous variables with non‐normal distributions were transformed using the natural logarithm (Ln) prior to analysis. ALT, alanine aminotransferase; AST, aspartate aminotransferase; cTnI, cardiac troponin I; NT‐proBNP, N‐terminal pro–B‐type natriuretic peptide; PaO2, partial pressure of oxygen.

Abbreviations: BMI, body mass index; BP, blood pressure; BUN, blood urea nitrogen; CHD, coronary heart disease; CI, confidence interval; D, dimer; DM, diabetes mellitus; HR, hazard ratio; LVEF, left ventricular ejection fraction; NT‐proBNP, N‐terminal pro–B‐type natriuretic peptide; PASP, pulmonary artery systolic pressure.

Based on this fully adjusted baseline model, additional multivariate analyses were conducted to evaluate the prognostic associations of inflammatory biomarkers (Supporting Information: Table S1). Several markers remained independently associated with increased risk of death: CRP (HR = 2.06, 95% CI: 1.55–2.73), WBC (HR = 1.38, 95% CI: 1.03–1.84), NEU (HR = 1.45, 95% CI: 1.10–1.91), MON (HR = 1.31, 95% CI: 1.01–1.70), and RDW (HR = 1.82, 95% CI: 1.40–2.36). Among composite indices, IBI (HR = 2.30, 95% CI: 1.67–3.17), NLR (HR = 1.81, 95% CI: 1.39–2.37), dNLR (HR = 1.80, 95% CI: 1.38–2.35), MLR (HR = 1.82, 95% CI: 1.40–2.38), NMLR (HR = 1.74, 95% CI: 1.33–2.27), PLR (HR = 2.25, 95% CI: 1.69–3.00), SII (HR = 1.70, 95% CI: 1.31–2.22), and SIRI (HR = 1.80, 95% CI: 1.38–2.34) were all significantly associated with mortality. By contrast, higher LYM was associated with a lower risk of death (HR = 0.69, 95% CI: 0.53–0.90). Notably, the IPS was independently associated with a higher risk of all‐cause mortality. Patients with high IPS (≥0.55) had a 2.55‐fold increased risk of death compared to those with low IPS (HR = 2.55, 95% CI: 1.92–3.38), after adjustment for the baseline clinical model.

### 3.5. Incremental Prognostic Value of IPS for All‐Cause Mortality

The prognostic relevance of the 15 inflammatory biomarkers was further evaluated using a RSF model. As shown in Figure 3A, VIMP analysis revealed that IPS, CRP, IBI, SIRI, and RDW contributed most to model performance. Consistently, the minimal depth analysis (Figure 3B) identified these variables as having the earliest splits in decision trees, indicating strong predictive capacity. A comparative plot (Figure 3C) demonstrated concordance between the two ranking methods, highlighting IPS as a particularly informative marker for mortality risk stratification in PE.

Figure 3Variable importance of inflammatory biomarkers for all‐cause mortality based on random survival forest (RSF) analysis. (a) Variable importance ranked by the variable importance (VIMP) measure, which quantifies the decrease in model prediction accuracy when each variable is permuted. (b) Variable importance ranked by minimal depth, representing the average depth of the first split for each variable across all trees; variables with smaller minimal depth are more predictive. (c) Comparison of rankings between the VIMP and minimal depth methods. The red dashed line represents perfect concordance. Variables above the line are ranked higher by minimal depth, while those below are ranked higher by VIMP.

**Figure:**
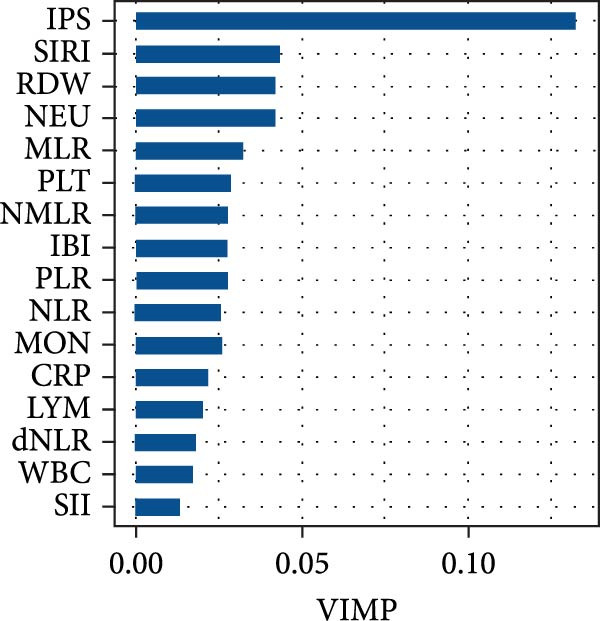
(a)

**Figure:**
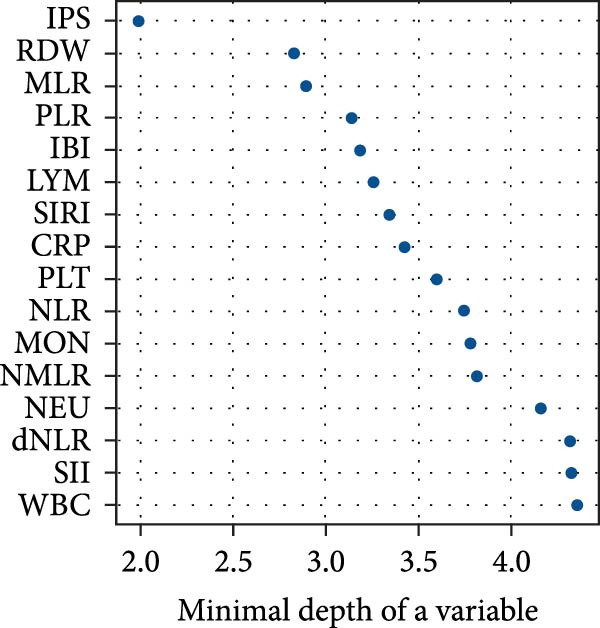
(b)

**Figure:**
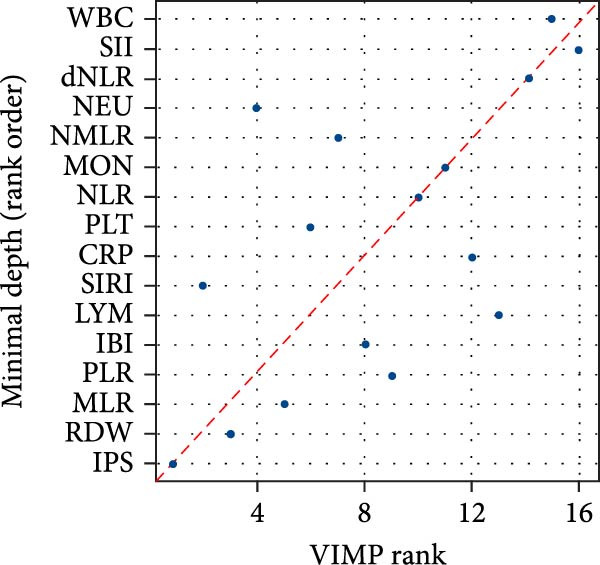
(c)

To assess the incremental prognostic value of IPS, time‐dependent ROC analysis was performed to compare the predictive accuracy of the baseline clinical model with and without IPS at 1, 3, and 5 years (Figure 4). The baseline model yielded AUCs of 0.819 (95% CI: 0.786–0.853), 0.820 (95% CI: 0.789–0.851), and 0.822 (95% CI: 0.788–0.855) at 1‐, 3‐, and 5‐year time points, respectively. With the addition of IPS, the corresponding AUCs increased to 0.879 (95% CI: 0.854–0.904), 0.886 (95% CI: 0.863–0.909), and 0.888 (95% CI: 0.863–0.913). These results confirm that IPS significantly enhances the discriminatory ability of the clinical model for predicting long‐term mortality in patients with PE.

Figure 4IPS improves the predictive performance of the basic clinical model for all‐cause mortality at 1, 3, and 5 years. Time‐dependent receiver operating characteristic (ROC) curves comparing the predictive accuracy of the basic model (blue line) and the basic model plus the Inflammatory Prognostic Score (IPS; red dashed line) at 1‐year (a), 3‐year (b), and 5‐year (c) time points. The basic model included the following covariates: age, length of hospital stay, deep vein thrombosis, diastolic blood pressure, body mass index, lactate, log‐transformed serum creatinine, blood urea nitrogen, log‐transformed D‐dimer, left ventricular ejection fraction, pulmonary artery systolic pressure, and CTPA‐based embolism location (main pulmonary artery).

**Figure:**
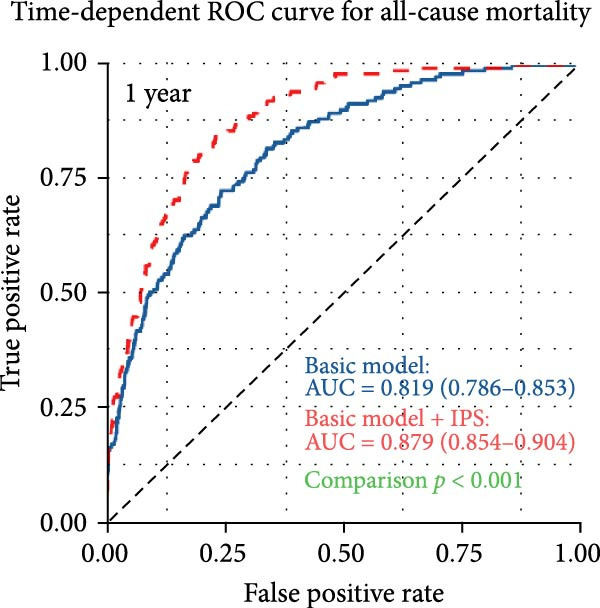
(a)

**Figure:**
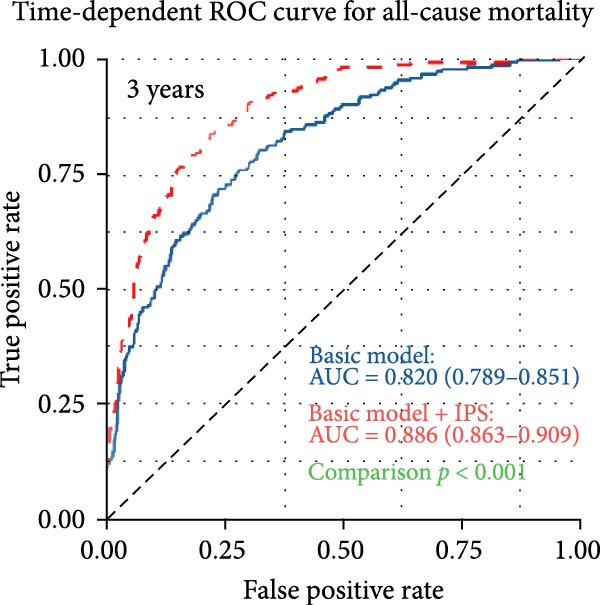
(b)

**Figure:**
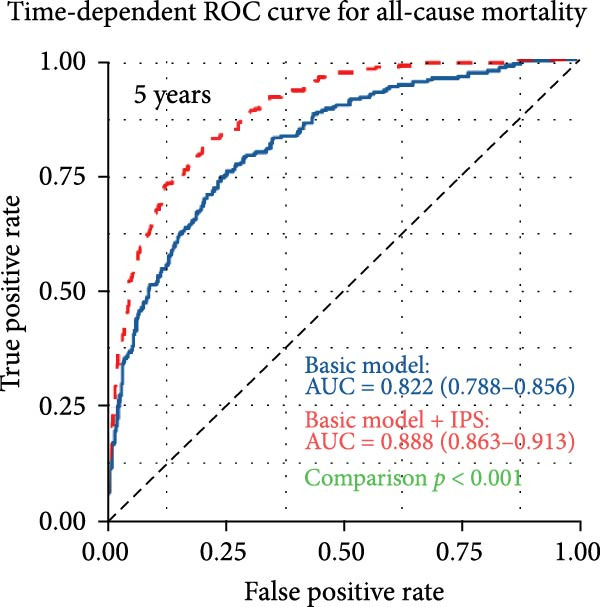
(c)

### 3.6. Sensitivity Analyses

Two prespecified sensitivity analyses were performed to assess the robustness of the association between the IPS and all‑cause mortality (Supporting Information: Table S2). After excluding participants with a history of malignancy (*n* = 147) or those with predefined inflammatory diseases (*n* = 621), high IPS remained significantly associated with an increased risk of death. The adjusted HRs were 2.55 (95% CI: 1.90–3.44; *p* < 0.001) and 2.27 (95% CI: 1.48–3.48; *p* < 0.001), respectively, confirming that the prognostic value of IPS was consistent and independent of malignancy or chronic inflammatory conditions.

## 4. Discussion

In this study, we developed and validated a novel IPS based on five routinely measured biomarkers (IBI, RDW, SIRI, MLR, and CRP) to predict long‐term all‐cause mortality in 1642 patients with acute PE. We found that high IPS levels were significantly associated with increased mortality risk among individuals with acute PE. In ROC analysis, IPS notably improved predictive accuracy with a significant increase in AUC values. Using a RSF model, both VIMP and minimal depth metrics consistently ranked IPS as the most influential predictors of mortality. Therefore, IPS could be used for more precise long‐term mortality prediction in patients with acute PE.

Several prior studies have identified individual inflammatory biomarkers as predictors of poor outcomes in acute PE, such as CRP, NLR, and RDW. IBI, a novel composite biomarker calculated from CRP and NEU and lLYM, has recently gained attention as a robust indicator of systemic inflammation [16]. A study involving 8827 participants from the NHANES database demonstrated that individuals with higher IBI values had significantly increased risks of all‐cause mortality [16]. In our study, IBI exhibited the strongest coefficient in the IPS formula and was identified as the most informative individual inflammatory predictor in both LASSO regression and RSF analyses. In a cohort of 162 patients with acute PE, RDW was significantly correlated with increased risk of all‐cause mortality [17]. RDW at a threshold of 18.15 demonstrated a sensitivity of 63% and a notably high specificity of 88% [17]. Jurin et al. [18] also found that patients with high on‐admission RDW values (>14.5% in men, >16.1% in women) had approximately a fourfold increased risk of 30‐day mortality compared to those with normal RDW. These findings suggest that RDW may add prognostic granularity beyond established clinical scores and support its inclusion in composite indices like the IPS. Recent studies have demonstrated that elevated SIRI is associated with adverse outcomes in PE [19]. A SIRI cutoff value of 2.87 was reported to identify high‐risk PE with an AUC of 0.624 [19]. MLR has also emerged as a relevant inflammatory marker in the context of cardiovascular and thromboembolic diseases. Although MLR has been less extensively studied in PE, recent evidence suggests its potential in identifying patients at increased risk [20]. Elevated CRP levels are known to reflect systemic inflammation and vascular injury, and have been linked to RV dysfunction [21]. Araz et al. [22] reported a significant association between elevated CRP levels and increased mortality, suggesting that serial measurements of CRP may be valuable in dynamic risk assessment. In line with this, other studies observed that patients classified as low risk exhibited significantly lower CRP levels compared to those in intermediate‐ and high‐risk categories [23]. We employed LASSO‐Cox regression to select five biomarkers with nonzero coefficients, including IBI, RDW, SIRI, MLR, and CRP. Each of these biomarkers demonstrated independent prognostic relevance. Its inclusion in our IPS model reflects this dual function, which could reinforce the idea that inflammatory markers like CRP can meaningfully contribute to more accurate and individualized risk stratification in patients with PE.

The systemic inflammatory biomarkers included in the score are inherently nonspecific and may not directly reflect vascular or pulmonary arterial inflammation specific to PE. Their circulating levels can be influenced by a variety of confounding factors, including concurrent infections, autoimmune conditions, malignancy, or other comorbidities commonly seen in hospitalized patients. Recent evidence has underscored the value of integrating multiple biomarkers into a unified prognostic model forPE [24]. A large registry‐based study involving over 60,000 haemodynamically stable patients with acute PE demonstrated that a multimarker calculator, which incorporated simplified PESI variables, natriuretic peptides, troponin, and presence of lower limb deep vein thrombosis, substantially improved risk stratification compared to the ESC model [24]. The multimarker tool significantly outperformed the ESC model in terms of discrimination for 30‐day all‐cause mortality, with a higher C‐statistic (0.79 vs. 0.56, *p* < 0.001) [24]. Importantly, the calculator’s positive predictive value for mortality at a >10% estimated risk threshold was more than threefold higher than that of the ESC model (15.7% vs. 5.0%, *p* < 0.001) [24]. Zhan et al. [25] developed a nomogram model incorporated age, right ventricular dysfunction (RVD), WBC, albumin‐to‐fibrinogen ratio (AFR), PNI, and SIRI, which demonstrated excellent prognostic utility in 30‐day mortality in patients with acute PE. These findings align with our study, which also emphasizes the prognostic advantages of combining multiple inflammatory markers into a single composite score. Much like the multimarker calculator, the IPS captures complementary dimensions of the pathophysiological response in PE, particularly the inflammatory burden. As nonspecific systemic indicators of inflammation, individual parameters are susceptible to variability and may be influenced by a wide range of physiological and pathological conditions [26]. Consequently, relying on single inflammatory markers can lead to inconsistent assessments due to the complexity and heterogeneity of confounding factors [26].

A wide range of prognostic markers have been identified in PE, which help in risk stratification and guiding clinical management. These include clinical scoring systems like the PESI, sPESI, and newer models such as the PUMCH rule have demonstrated utility in predicting short‐term mortality [27, 28]. However, few models have shown robust performance in long‐term outcome prediction. In our study, the IPS showed strong predictive value for long‐term all‐cause mortality, with time‐dependent AUCs of 0.756, 0.768, and 0.773 at 1, 3, and 5 years, respectively. Patients with a high IPS (≥0.55) had a significantly higher risk of mortality compared to those with low IPS (<0.55), with an adjusted HR of 2.55 (95% CI: 1.92–3.38, *p* < 0.001). Furthermore, when IPS was incorporated into a baseline clinical model, the 3‐year AUC improved from 0.820 to 0.886 (*p* < 0.001, DeLong test). Incorporating systemic inflammatory markers into existing risk assessment frameworks might be helpful for more precise and individualized prognosis in PE.

Incorporating inflammatory markers into prognostic models may provide additional insights into the biological mechanisms underlying PE‐related complications. Inflammation is not merely a consequence but an integral component of the disease process. During the processes of thrombus formation and resolution, dynamic changes occur in the composition and activity of various inflammatory cells [29, 30]. Neutrophils, monocytes, lymphocytes, and macrophages are recruited to the thrombus site, which contribute to both procoagulant activity and subsequent fibrinolysis and tissue repair [30]. These cellular shifts reflect the complex interplay between coagulation and inflammation. In the acute phase of PE, the release of proinflammatory cytokines and acute‐phase proteins such as CRP, interleukin‐6 (IL‐6), and tumor necrosis factor‐alpha (TNF‐*α*) promotes a procoagulant state by upregulating tissue factor expression [31]. These mediators enhance the expression of tissue factor on monocytes and endothelial cells, and promote thrombin generation and contribute to a hypercoagulable state [32]. Meanwhile, endogenous agents like polyphosphates and bradykinin activate the contact system, which further stimulate the intrinsic and extrinsic coagulation pathways [32]. Collectively, these mechanisms might illustrate how inflammatory responses actively sustain and propagate thrombosis.

In this study, we developed and validated a novel tool integrating routinely available inflammatory biomarkers to predict mortality in patients with acute PE. The IPS was developed using LASSO‐Cox regression, which reduced overfitting and selected only the most informative variables. Moreover, markers such as IBI and RDW were shown to be independently associated with mortality and had high specificity. This ensures a parsimonious yet powerful prognostic tool. the IPS demonstrated strong and consistent prognostic performance across multiple analytical methods. Moreover, the biomarkers used in IPS are routinely measured in standard laboratory panels, which could make the score widely accessible and economically feasible in clinical settings. Further multicenter studies are warranted to validate the generalizability of IPS and assess its utility in guiding personalized management strategies in acute PE.

However, several limitations should be acknowledged. First, although a thorough review of medical records was performed, reliance on existing documentation may introduce biases related to incomplete or inconsistent data entry. Second, although the IPS integrated multiple markers to mitigate the limitations of single‐variable models, the potential for residual confounding remains due to unmeasured or overlapping systemic factors. Third, the study was conducted retrospectively at a single health care center, which may limit the generalizability of the findings. External validation in prospective, multicenter cohorts is needed to confirm the reproducibility and clinical applicability of the IPS. Fourth, the use of laboratory parameters obtained at a single time point may not fully capture the dynamic nature of inflammation during the course of PE, and future studies should explore the prognostic value of serial biomarker measurements. Final, because of the retrospective design and constraints of the electronic health records, we could not reliably distinguish first episode from recurrent PE across all participants, nor could we comprehensively capture every potentially PE‐associated comorbidity beyond those consistently documented. These data limitations may have introduced unmeasured heterogeneity and residual confounding.

## 5. Conclusion

In this retrospective cohort study of patients with PE, we developed an IPS based on five routinely available biomarkers: IBI, RDW, SIRI, MLR, and CRP. The IPS was independently associated with all‐cause mortality and significantly enhanced the predictive performance of an established clinical model. Owing to its simplicity, accessibility, and interpretability, the IPS may serve as a valuable tool for early risk stratification and long‐term prognostic assessment in patients with PE. Further prospective studies and external validation are needed to confirm its generalizability and clinical applicability.

## Ethics Statement

The study protocol was approved by the Independent Ethics Committee of the First Affiliated Hospital of Ningbo University (No. 2024‐005RS) and the Affiliated People Hospital of Ningbo University (No. 2025‐027). This study was conducted in accordance with the Declaration of Helsinki. The requirement for written informed consent was exempted because of the retrospective nature.

## Disclosure

All authors approved the final draft of the manuscript for publication. All authors have approved the final version of the manuscript for publication and have agreed to be responsible for the research presented.

## Conflicts of Interest

The authors declare no conflicts of interest.

## Author Contributions

Ning Zhu, Chao Cao and Limin Chen contributed to hypothesis development and manuscript preparation. Lei Zhang, Jiabao Wu, Fei Ye and Nanding Yu undertook data analyses. Ning Zhu and Lei Zhang drafted and revised the manuscript.

## Funding

This study was supported by the Medical and Health Science and Technology Project of Zhejiang Province (Grant 2024KY1546) and the Fifth Batch of Young Technical Backbone Talents Project of the Ningbo Municipal Health Commission (to Ning Zhu). This work was supported in part by Grant 2023KY253 from Health Commission of Zhejiang Province.

## Supporting Information

Additional supporting information can be found online in the Supporting Information section.

## Supporting information


**Supporting Information** Figure S1: Time‐dependent receiver operating characteristic (ROC) curves for 15 inflammatory biomarkers in predicting all‐cause mortality. Figure S2: Kaplan–Meier survival curves for all‐cause mortality stratified by 15 inflammatory biomarkers using optimal cut‐off values derived from 3‐year time‐dependent ROC analysis. Figure S3: Pairwise Spearman correlation matrix among 15 inflammatory biomarkers. Table S1: Univariate and multivariate stepwise Cox regression analysis of all‐cause mortality in patients with pulmonary embolism. Table S2: Association of the inflammatory prognostic score (IPS) with all‐cause mortality after excluding participants with a history of malignancy or inflammatory disease at baseline in patients with pulmonary embolism.

## Data Availability

The data that support the findings of this study are available from the corresponding author upon reasonable request.
