# Supplementary material for: Development and Evaluation of an Innovative Inflammatory Prognostic Score for Predicting Long‐Term Mortality in Patients With Pulmonary Embolism
Source: Mediators Inflamm. 2025 Dec 22;2025:6325915. doi: 10.1155/mi/6325915 (PMC12767386; doi:10.1155/mi/6325915)
Supplement: Supplementary file 1 — Supporting Information Figure S1: Time‐dependent receiver operating characteristic (ROC) curves for 15 inflammatory biomarkers in predicting all‐cause mortality. Figure S2: Kaplan–Meier survival curves for all‐cause mortality stratified by 15 inflammatory biomarkers using optimal cut‐off values derived from 3‐year time‐dependent ROC analysis. Figure S3: Pairwise Spearman correlation matrix among 15 inflammatory biomarkers. Table S1: Univariate and multivariate stepwise Cox regression analysis of all‐cause mortality in patients with pulmonary embolism. Table S2: Association of the inflammatory prognostic score (IPS) with all‐cause mortality after excluding participants with a history of malignancy or inflammatory disease at baseline in patients with pulmonary embolism. [file MI-2025-6325915-s001.docx]

**Supplementary Material**

**Development and Evaluation of an Innovative Inflammatory Prognostic Score for Predicting Long-Term Mortality in Patients with Pulmonary Embolism**

**
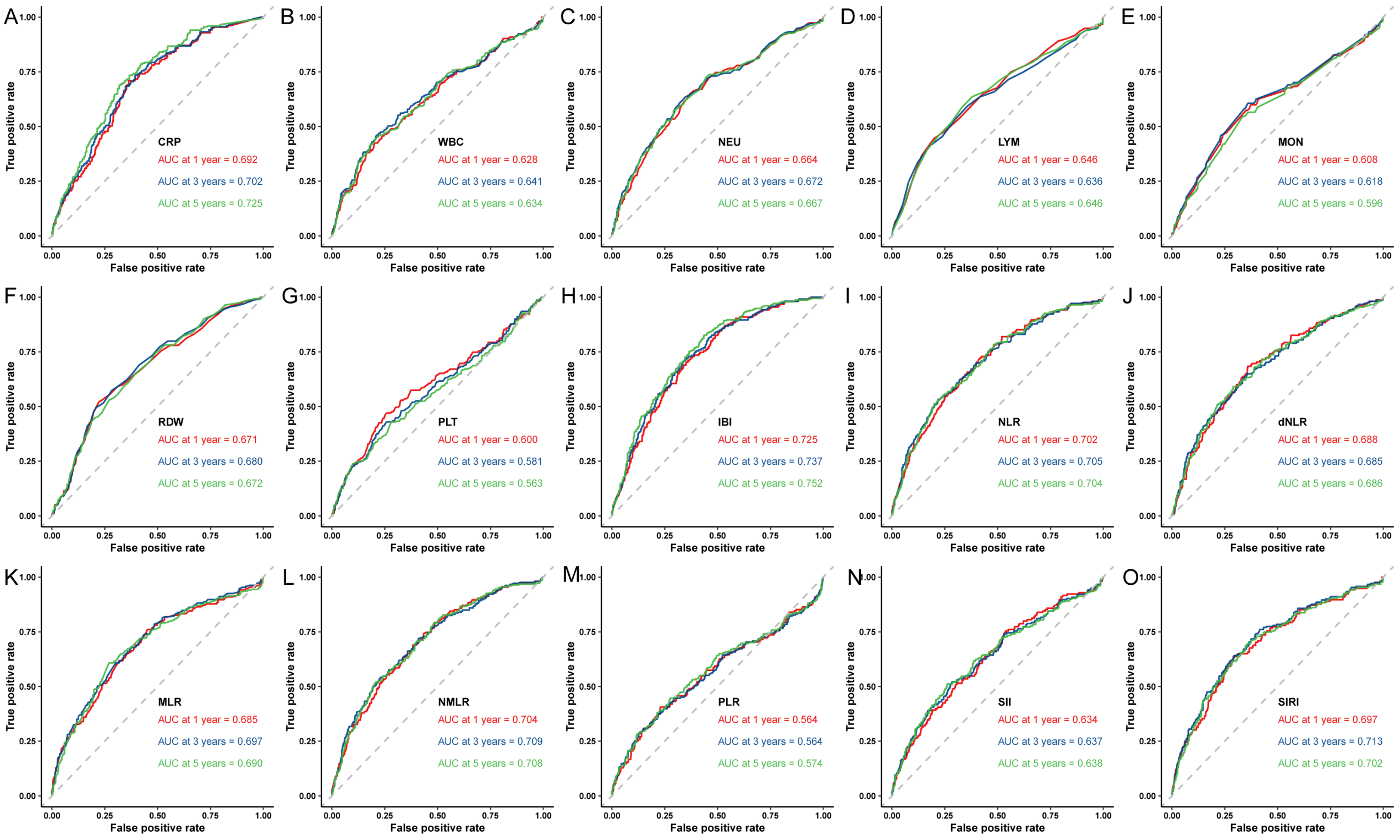
**

**Figure S1.** Time-dependent receiver operating characteristic (ROC) curves for 15 inflammatory biomarkers in predicting all-cause mortality. ROC curves were plotted at 1 year (red), 3 years (blue), and 5 years (green) follow-up intervals, and the corresponding area under the curve (AUC) values are displayed in each panel. The diagonal dashed line represents the reference line (AUC = 0.5), indicating no discriminative ability. Given the median follow-up duration of 43.87 ± 27.25 months, the optimal cut-off values for each biomarker were determined based on the maximum Youden index at the 3-year time point.

Panels A–O represent the following biomarkers: (A) C-reactive protein (CRP), (B) white blood cell count (WBC), (C) neutrophil count (NEU), (D) lymphocyte count (LYM), (E) monocyte count (MON), (F) red cell distribution width (RDW), (G) platelet count (PLT), (H) inflammatory burden index (IBI), (I) neutrophil-to-lymphocyte ratio (NLR), (J) derived neutrophil-to-lymphocyte ratio (dNLR), (K) monocyte-to-lymphocyte ratio (MLR), (L) neutrophil–monocyte-to-lymphocyte ratio (NMLR), (M) platelet-to-lymphocyte ratio (PLR), (N) systemic immune-inflammation index (SII), and (O) systemic inflammation response index (SIRI).

**
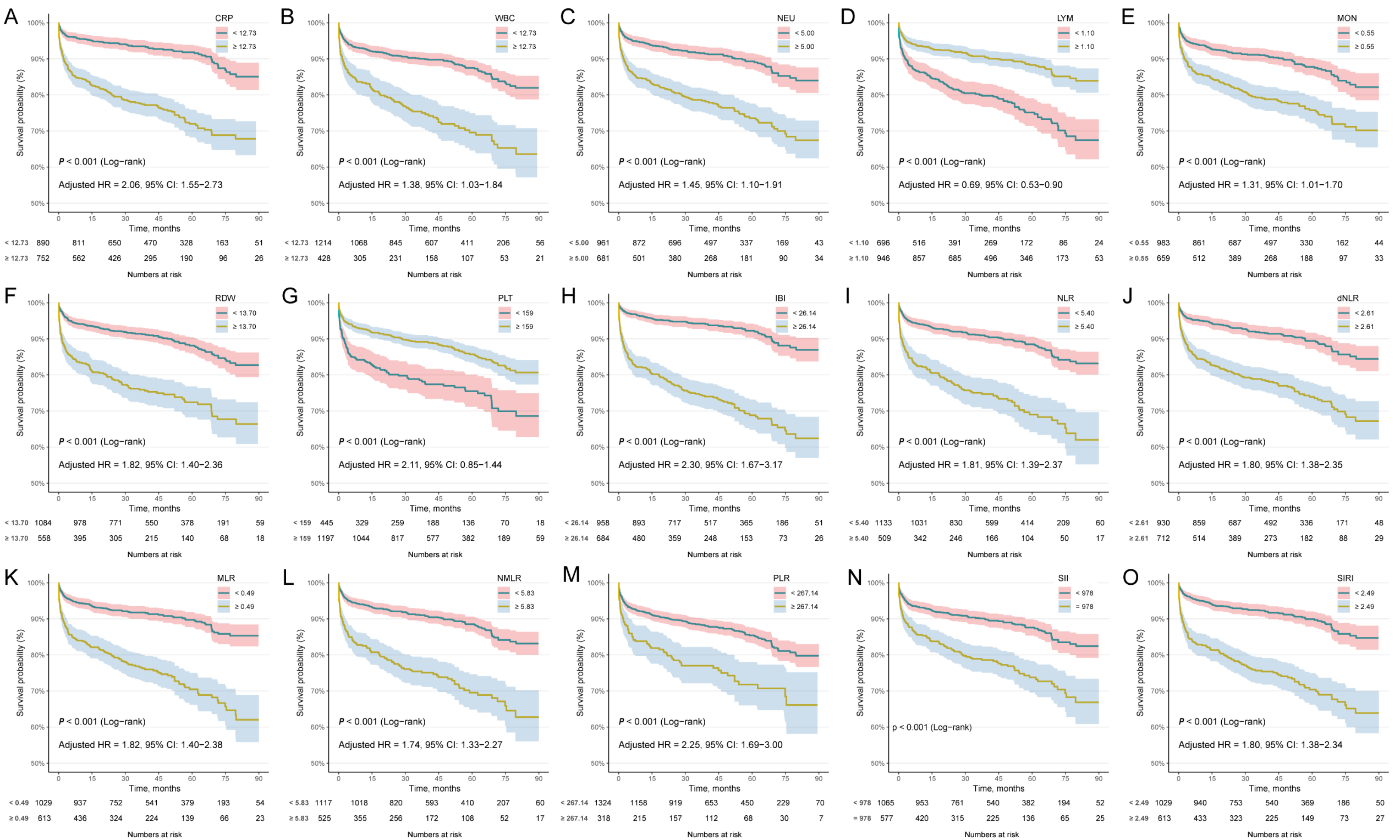
**

**Figure S2.** Kaplan–Meier survival curves for all-cause mortality stratified by 15 inflammatory biomarkers using optimal cut-off values derived from 3-year time-dependent ROC analysis. Each panel (A–O) represents a separate biomarker: (A) C-reactive protein (CRP), (B) white blood cell count (WBC), (C) neutrophil count (NEU), (D) lymphocyte count (LYM), (E) monocyte count (MON), (F) red cell distribution width (RDW), (G) platelet count (PLT), (H) inflammatory burden index (IBI), (I) neutrophil-to-lymphocyte ratio (NLR), (J) derived neutrophil-to-lymphocyte ratio (dNLR), (K) monocyte-to-lymphocyte ratio (MLR), (L) neutrophil–monocyte-to-lymphocyte ratio (NMLR), (M) platelet-to-lymphocyte ratio (PLR), (N) systemic immune-inflammation index (SII), and (O) systemic inflammation response index (SIRI). Participants were categorized into high and low groups based on biomarker-specific optimal thresholds determined by the maximum Youden index. Survival differences were assessed using the log-rank test. Adjusted hazard ratios (HRs) with 95% confidence intervals (CIs) were estimated using Cox proportional hazards models. The multivariable models were adjusted for key clinical covariates, including age, length of hospital stay, history of deep vein thrombosis, diastolic blood pressure, body mass index (BMI), lactate level, natural logarithm of serum creatinine, blood urea nitrogen (BUN), natural logarithm of D-dimer, left ventricular ejection fraction (LVEF), pulmonary artery systolic pressure (PASP), and main pulmonary artery diameter. Shaded regions around survival curves indicate 95% CIs.

**
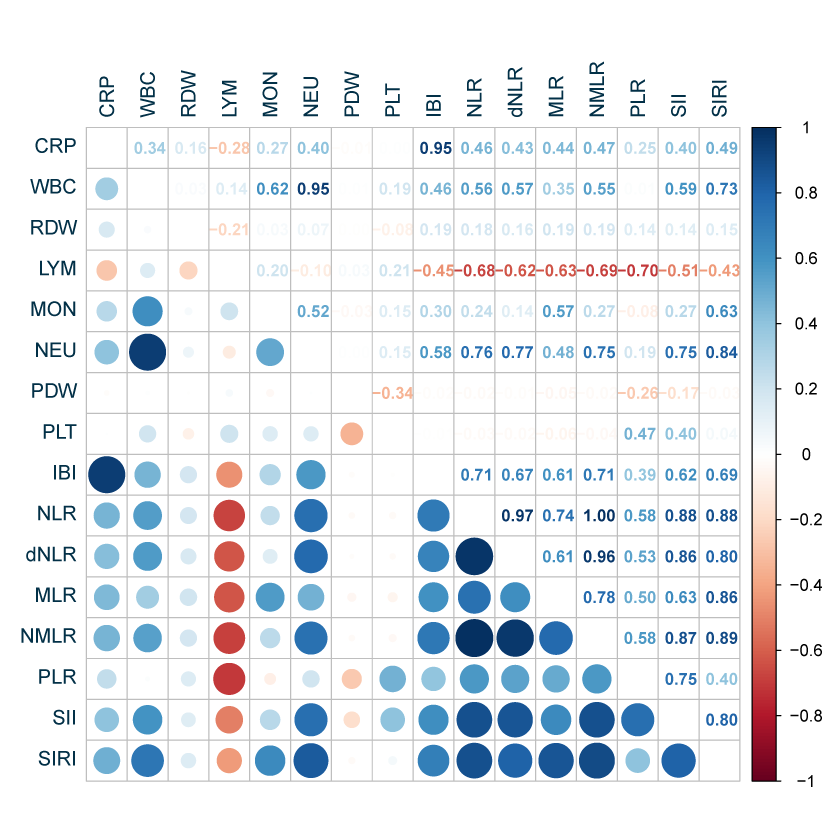
**

**Figure S3.** Pairwise Spearman correlation matrix among 15 inflammatory biomarkers. The correlation coefficients are shown within the upper triangle of the matrix, while the corresponding color intensity and size of the circles in the lower triangle reflect the strength and direction of the correlations (blue: positive; red: negative).

**Table S1.** Univariate and multivariate stepwise Cox regression analysis of all-cause mortality in patients with pulmonary embolism.

|  | Univariate analysis | |  | Multivariate analysis | |
| --- | --- | --- | --- | --- | --- |
|  | HR (95%CI) | *P* value |  | HR (95%CI) | *P* value |
| CRP ≥ 12.73, mg/L | 3.23 (2.48-4.20) | <0.001 |  | 2.06 (1.55-2.73) | <0.001 |
| WBC ≥ 8.91, 10^9^/L | 2.55 (2.00-3.26) | <0.001 |  | 1.38 (1.03-1.84) | 0.029 |
| NEU ≥ 5.00, 10^9^/L | 2.59 (2.02-3.32) | <0.001 |  | 1.45 (1.10-1.91) | 0.008 |
| LYM ≥ 1.10, 10^9^/L | 0.44 (0.35-0.57) | <0.001 |  | 0.69 (0.53-0.90) | 0.005 |
| MON ≥ 0.55, 10^9^/L | 2.13 (1.67-2.72) | <0.001 |  | 1.31 (1.01-1.70) | 0.041 |
| RDW ≥ 13.70, % | 2.62 (2.06-3.34) | <0.001 |  | 1.82 (1.40-2.36) | <0.001 |
| PLT ≥ 159.00, 10^9^/L | 0.51 (0.40-0.66) | <0.001 |  | 1.11 (0.85-1.44) | 0.445 |
| IBI ≥ 62.58, mg/L | 4.12 (3.04-5.58) | <0.001 |  | 2.30 (1.67-3.17) | <0.001 |
| NLR ≥ 5.40 | 2.84 (2.23-3.62) | <0.001 |  | 1.81 (1.39-2.37) | <0.001 |
| dNLR ≥ 2.61 | 2.68 (2.08-3.44) | <0.001 |  | 1.80 (1.38-2.35) | <0.001 |
| MLR ≥ 0.49 | 3.01 (2.35-3.86) | <0.001 |  | 1.82 (1.40-2.38) | <0.001 |
| NMLR ≥ 5.83 | 2.78 (2.18-3.55) | <0.001 |  | 1.74 (1.33-2.27) | <0.001 |
| PLR ≥ 267.14 | 2.02 (1.55-2.65) | <0.001 |  | 2.25 (1.69-3.00) | <0.001 |
| SII ≥ 298.09, 10^9^/L | 2.19 (1.72-2.79) | <0.001 |  | 1.70 (1.31-2.22) | <0.001 |
| SIRI ≥ 2.49, 109/L | 3.13 (2.44-4.01) | <0.001 |  | 1.80 (1.38-2.34) | <0.001 |
| IPS ≥ 0.55 | 4.20 (3.22-5.48) | <0.001 |  | 2.55 (1.92-3.38) | <0.001 |

Abbreviation: CRP, C-reactive protein; WBC, white blood cell count; NEU, neutrophil count; LYM, lymphocyte count; MON, monocyte count; RDW, red cell distribution width; PLT, platelet count; IBI, inflammatory burden index; NLR, neutrophil-to-lymphocyte ratio; dNLR, derived neutrophil-to-lymphocyte ratio; MLR, monocyte-to-lymphocyte ratio; NMLR, neutrophil-monocyte-to-lymphocyte ratio; PLR, platelet-to-lymphocyte ratio; SII, systemic immune-inflammation index; SIRI, systemic inflammation response index; IPS, inflammation prognostic score.

**Table S2.** Association of the inflammatory prognostic score (IPS) with all-cause mortality after excluding participants with a history of malignancy or inflammatory disease at baseline in patients with pulmonary embolism.

|  | Univariate analysis | |  | Multivariate analysis† | |
| --- | --- | --- | --- | --- | --- |
|  | HR (95%CI) | *P* value |  | HR (95%CI) | *P* value |
| Primary analysis (full cohort) (n=1642) |  |  |  |  |  |
| Low IPS | 1.00 [Reference] |  |  | 1.00 [Reference] |  |
| High IPS | 4.20 (3.22-5.48) | <0.001 |  | 2.55 (1.92-3.38) | <0.001 |
| Excluding malignancy history (n=1495) |  |  |  |  |  |
| Low IPS | 1.00 [Reference] |  |  | 1.00 [Reference] |  |
| High IPS | 4.32 (3.26-5.72) | <0.001 |  | 2.55 (1.90-3.44) | <0.001 |
| Excluding inflammatory disease set (n=1021) * | |  |  |  |  |
| Low IPS | 1.00 [Reference] |  |  | 1.00 [Reference] |  |
| High IPS | 3.65 (2.48-5.37) | <0.001 |  | 2.27 (1.48-3.48) | <0.001 |

*Inflammatory disease set: To minimize confounding from systemic inflammatory states, we predefined an inflammatory disease set comprising: autoimmune/connective-tissue disorders (e.g., rheumatoid arthritis, systemic lupus erythematosus, or vasculitides), inflammatory bowel disease, autoimmune or subacute thyroiditis, myeloproliferative/auto-inflammatory disorders, and active infection within 30 days or admission CRP >20 mg/L with supportive clinical evidence. Recent (≤3 months) systemic corticosteroid or immunosuppressant therapy for an inflammatory indication was also considered indicative of active inflammatory disease. Conditions were ascertained from electronic medical records (physician documentation, discharge diagnoses/problem lists, medication records, and relevant laboratory data).

†Multivariable Cox regression model adjusted for age, length of hospital stay, deep vein thrombosis, diastolic blood pressure, body mass index, lactate, log-transformed serum creatinine, blood urea nitrogen, log-transformed D-dimer, left ventricular ejection fraction, pulmonary artery systolic pressure, and CTPA-based embolism location (main pulmonary artery).
